# Vers l’élimination de la maladie du sommeil

**DOI:** 10.48327/mtsi.v3i1.2023.317

**Published:** 2023-02-10

**Authors:** Philippe Solano, Fabrice Courtin, Dramane Kaba, Camara, Moïse Kagbadouno, Jean-Baptiste Rayaisse, Vincent Jamonneau, Bruno Bucheton, Jean-Mathieu Bart, Sophie Thevenon, Veerle Lejon

**Affiliations:** 1 Université Montpellier, Institut de recherche pour le développement (IRD), Centre de coopération internationale en recherche agronomique pour le développement (CIRAD), UMR Intertryp Montpellier, France; 2 Représentation IRD Ouagadougou, Burkina Faso; 3 Institut Pierre Richet Bouaké, Institut national de santé publique, Côte d'Ivoire; 4 Programme national de lutte contre les maladies tropicales négligées – Prise en charge des cas (PNLMTN-PCC), Ministère de la Santé, Conakry, Guinée; 5 Centre international de recherche-développement sur l’élevage (CIRDES), Bobo-Dioulasso, Burkina Faso; * Actes du Colloque – Centenaire de la mort d'Alphonse Laveran. 24 novembre 2022, Paris / Proceedings of the Conference – Centenary of the death of Alphonse Laveran. 24 November 2022, Paris

**Keywords:** Alphonse Laveran, Trypanosomiase humaine africaine, Diagnostic, Traitement, Lutte antivectorielle, Élimination, Alphonse Laveran, Human African Trypanosomiasis, Diagnostic, Treatment, Vector control, Elimination

## Abstract

La maladie du sommeil, ou trypanosomiase humaine africaine (THA), est due à *Trypanosoma brucei* transmis par *Glossina* spp ou mouche tsé-tsé. Après avoir ravagé l'Afrique subsaharienne dans la première moitié du XX^e^ siècle, comme en témoigne l'extraordinaire ouvrage d'Alphonse Laveran et Félix Mesnil en 1912 (*Trypanosomes et trypanosomiases*), la THA, maladie tropicale négligée mortelle, dite « du bout de la piste » et pour laquelle il n'existe pas de vaccin, semble aujourd'hui à portée de main de l’élimination. Nous passons en revue les stratégies, activités et outils qui ont permis cette forte réduction du fardeau sanitaire pour les populations d'Afrique subsaharienne: environ 300 000 cas estimés dans les années 1990 contre moins de 1000 cas rapportés annuellement depuis 2018. La lutte contre la maladie du sommeil consiste principalement à dépister et traiter les cas ainsi qu’à lutter contre la glossine vectrice pour casser le cycle de transmission. En passant en 30 ans d'un contexte épidémique à un contexte d’élimination, la maladie du sommeil a subi une transition épidémiologique sans précédent à laquelle les stratégies et les outils de lutte ont dû s'adapter. Nous montrons comment la recherche a soutenu ces efforts et examinons certains des défis restants pour rendre effective et durable son élimination.

## Abstract (en)

Human African Trypanosomiasis (HAT) is caused by *Trypanosoma brucei* which is transmitted by the tsetse fly insect vector (*Glossina* spp). It is one of the 20 Neglected Tropical Diseases (NTD) listed by the WHO. These diseases affect the poorest and most vulnerable communities, for which the WHO has established a dedicated 2021-2030 roadmap. At the time of Alphonse Laveran, HAT devastated the African continent. In the 1960s, the disease was nearly under control, but it strongly re-emerged in the 1990s. A coordinated effort of all stakeholders, with national control programs as the main actors, a strong contribution of research and important donations by the private sector, allowed to decrease the HAT burden significantly. Since 2018, less than 1000 cases are detected annually. We here review new diagnostics, treatments and vector control tools that have been implemented jointly and successfully in several endemic countries.

The next key challenge will be to sustain the gains. Newly emerging research questions include long-term carriage of trypanosomes and adaptation of tools to low prevalence contexts. Challenges out of the research area comprise the continued need of funding, maintenance of dedicated human resources, and the key question of access. Sustainable elimination as “interruption of transmission”, which is the 2030 NTD roadmap target, can be reached, if these challenges are solved. We stress the importance of continuing to combine the efforts in the fight against the disease, because sustainable elimination of HAT is the best long-term prevention strategy against re-emergence. As such, HAT elimination can serve as an example for other infectious diseases.

## Introduction

En cet hommage à Alphonse Laveran, il n'est pas inutile de rappeler le fabuleux ouvrage publié par Laveran et Mesnil en 1912, *Trypanosomes et trypanosomiases* [12]. Ce document, qui peut être téléchargé gratuitement sur www.biodiversitylibrary.org/item/15534, reste une source d'inspiration extraordinaire avec des observations et questionnements toujours d'actualité. À titre d'exemple le texte suivant accompagne la description de la carte des foyers de maladie du sommeil connus à l’époque, suite à la mission de Messieurs Martin, Lebœuf et Roubaud: « En résumé, sur la côte ouest de l'Afrique, depuis le Sénégal jusqu’à Saint-Paul-de-Loanda, la plupart des régions sont atteintes mais, en général, l'endémie n'y exerce pas de grands ravages; au contraire la maladie du sommeil sévit avec force au Congo français, dans certaines parties du Congo belge et dans l'Ouganda; elle est dans ces pays en voie d'extension. » Par ces mots, Laveran et Mesnil décrivaient les prémices d'une des plus grandes épidémies qui était en train de naître sur le continent africain, et qui impacta considérablement le peuplement de cette région du monde [4].

Il est clairement énoncé en préambule, et conformément au titre de cette présentation, que nous ne parlerons pas dans ce travail sur la THA « d’éradication^1^1Définitions.**Élimination comme problème de santé publique**: réduction de l'incidence, de la prévalence, de la mortalité, du fardeau ou de l'impact de la maladie à un niveau localement acceptable à la suite d'efforts délibérés. Des mesures d'intervention continues sont nécessaires pour maintenir la réduction jusqu’à un seuil pouvant être fixé où la maladie n'est plus considérée comme un problème de santé publique.**Élimination avec interruption de la transmission:** réduction à zéro de l'incidence de la maladie dans une zone géographique définie à la suite d'efforts délibérés. Des mesures continues pour empêcher le rétablissement de la transmission peuvent être nécessaires.**Éradication:** réduction permanente à zéro de l'incidence mondiale de la maladie à la suite d'efforts délibérés. Les mesures de lutte pour empêcher le rétablissement de la transmission ne sont plus nécessaires. », mais bien « d’élimination », conformément aux définitions et cibles de la feuille de route de l'Organisation mondiale de la santé (OMS) [19].

### La THA, une maladie tropicale négligée

Les maladies tropicales négligées (MTN) regroupent 20 maladies, pour la plupart infectieuses et dues à une variété d'agents pathogènes, dont certains sont transmis par des vecteurs: virus, bactéries, champignons, parasites^2^2Liste des 20 MTN: helminthiases transmises par le sol, filariose lymphatique, onchocercose, dracunculose, trématodoses d'origine alimentaire, bilharziose, échinococcose, cysticercose (MTN dues aux helminthes); trypanosomiase humaine africaine, leishmanioses, maladie de Chagas (MTN dues aux protozoaires); rage, dengue (Zika, chikungunya) (MTN dues à des virus); ulcère de Buruli, trachome, lèpre, pian (MTN dues à des bactéries); gale, envenimations par morsure de serpent, mycétome (MTN dues à d'autres causes).. Elles ont pour point commun d'atteindre les communautés les plus vulnérables (« les plus pauvres des pauvres ») sur tous les continents, qui représentent presque 2 milliards de personnes dans 150 pays. Ces maladies, prises ensemble, sont regroupées au sein d'un département au sein de l'OMS, et font l'objet de donations de médicaments de la part des compagnies pharmaceutiques. Les MTN apparaissent dans la cible 3.3 des objectifs de développement durable, ont fait l'objet d'une résolution spécifique de l'Organisation internationale de la francophonie en 2018, et d'un groupe dédié du G7. Récemment, l'OMS a publié la « feuille de route 2021-2030 des MTN » en définissant des cibles d’éradication (pour 2 maladies, le pian et la dracunculose), d’élimination avec interruption de la transmission, d’élimination comme problème de santé publique, et de contrôle [19]. Des progrès récents ont été réalisés et sont documentés avec par exemple, 43 pays ayant éliminé au moins une MTN en 2021.

### La THA, deux maladies, une approche « One Health » évidente

La Trypanosomiase humaine africaine (THA) ou maladie du sommeil, qui fait partie des MTN, est due à un parasite extracellulaire, protozoaire de la famille des Trypanosomatidae de l'espèce *Trypanosoma brucei*, transmis à l'homme par la piqûre infectante d'un vecteur, la mouche tsé-tsé (Diptera: Glossinidae). Cette transmission vectorielle est limitée à l'Afrique subsaharienne qui constitue la limite de l'aire de répartition du vecteur. Chez l'homme, après une première phase hémato-lymphatique, le trypanosome envahit le système nerveux central pour donner la seconde phase, méningo-encéphalitique. En l'absence de traitement, la THA est considérée comme toujours fatale. Cette endémie regroupe en réalité deux formes différentes:

La THA à *Trypanosoma brucei gambiense* regroupe environ 98% des cas rapportés, elle sévit en Afrique de l'Ouest Centrale, est considérée comme une maladie essentiellement humaine avec possible implication d'un réservoir animal (outre le vecteur qui en constitue un) dont l'importance dans la transmission reste à établir. L'infection à *T. b. gambiense* montre chez l'homme des parasitémies sanguines faibles et fluctuantes, et cette forme chronique de la maladie dure plusieurs années.

La THA à *T. b. rhodesiense* constitue seulement 2% des cas mais semble plus difficile à contrôler car il s'agit d'une zoonose, localisée en Afrique de l'Est et australe, avec des parasitémies sanguines fortes et une issue fatale très rapide chez l'homme (quelques semaines).

LA THA semblait avoir été contrôlée dans les années 1960, mais a fortement ré-émergé avec une estimation autour de 300 000 cas dans les années 1990 suite à la baisse des activités de lutte et aux profonds bouleversements survenus du fait de la croissance démographique et ses conséquences. C'est une maladie parasitaire ne faisant pas ou peu l'actualité, qui touche les populations les plus reculées d'Afrique rurale même si le phénomène d'urbanisation a permis à des foyers urbains et péri-urbains de se développer également. Qualifiée de « maladie du bout de la piste », elle est particulièrement stigmatisante et nécessite une prise en charge individuelle des cas par des personnels de santé spécifiquement formés aux techniques complexes de diagnostic et de traitement.

## État Des Lieux Du Diagnostic, Du Traitement Et De La Lutte Antivectorielle

Pour lutter contre la THA, il n'existe aucun vaccin et aucune chimioprophylaxie. Les activités de lutte pour interrompre la transmission en soignant les malades reposent donc, dans un scénario idéal, sur une combinaison d'activités médicales (diagnostic + traitement) et de lutte antivectorielle.

### Diagnostic

**Figure 1:**
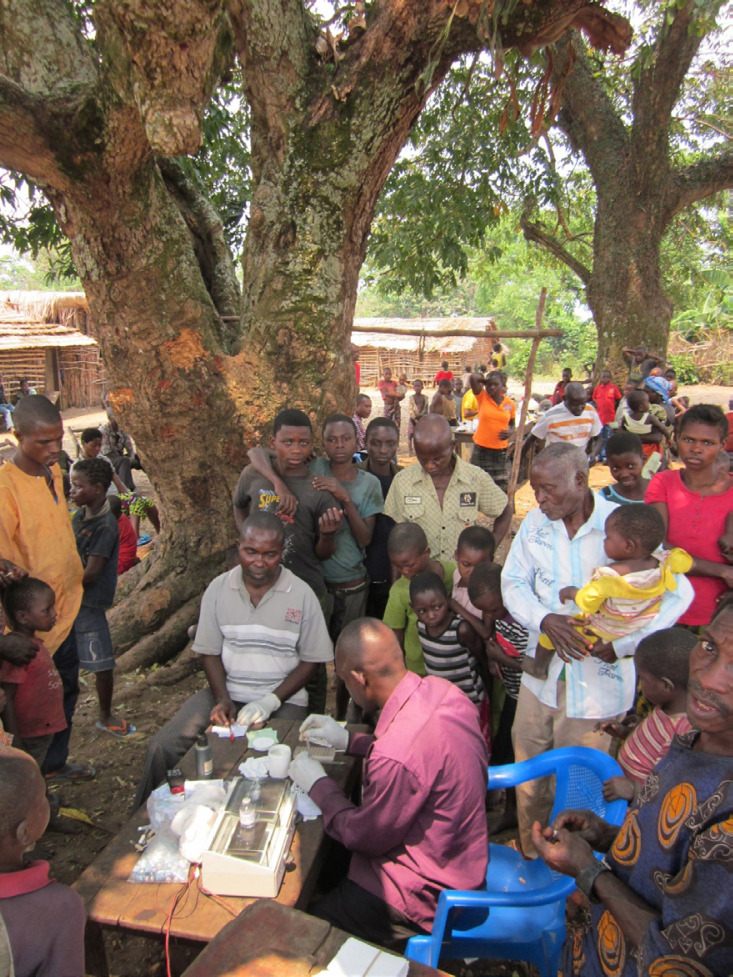
Prospection médicale, 2013, province Kwilu, RDC (crédit photo: Veerle Lejon) Medical prospection 2013, Kwilu Province, DRC (photo credit: Veerle Lejon)

Le diagnostic de la THA due à *T. b. gambiense* implique des stratégies différentes avec des outils adaptés. On distingue le « dépistage actif », à l'image des prospections médicales que réalisaient les équipes du Dr Jamot au siècle dernier, où c'est l’équipe médicale qui va à la rencontre de la population à risque qui a été informée et sensibilisée en amont. Plusieurs centaines de personnes peuvent être testées « sous le baobab » par des outils de dépistage de masse comme le CATT (Card Agglutination Test for Trypanosomiasis) après prélèvement d'une goutte de sang par piqûre au bout du doigt (Fig. 1).

Bien qu'existant toujours, cette stratégie est moins utilisée dans un contexte de basse prévalence pour des raisons évidentes et diverses (désintérêt de la population, coût, efficacité moindre, etc.), mais conserve son intérêt dans le but de connaître la prévalence de l'endémie. L'on pratique de plus en plus des stratégies de dépistage « réactif ciblé » ou en « porte-à-porte », qui se mettent en place au voisinage d'un cas récemment diagnostiqué en testant tout son entourage, mais pas au-delà [9, 11]. Enfin, le dépistage en poste fixe, ou « passif », consiste à établir une capacité de diagnostic de la THA dans des centres de santé périphériques; c'est alors la population qui va vers le système de santé. Pour ces deux dernières stratégies sont utilisés des tests de diagnostic de type individuel comme des « tests de diagnostic rapides » (TDR) mis au point ces dernières années [2, 3, 9].

Le diagnostic de la THA consiste donc en une procédure à plusieurs étapes: d'abord une suspicion (clinique et/ou sérologique), puis une confirmation parasitologique encore considérée comme obligatoire dans beaucoup de pays (et recommandée par l'OMS) avant de traiter un patient.

Les tests de diagnostic les plus utilisés en routine sont donc: le CATT pour les prospections médicales actives (ne convient pas pour un diagnostic individuel), et les TDR effectués sur sang. Sachant que ces deux outils, mis au point à partir des mêmes antigènes, détectent des anticorps anti-trypanosomes. Seuls deux TDR sont commercialisés actuellement. La confirmation parasitologique par examen microscopique, autre clin d'oeil à Laveran, peut être faite à partir d'un prélèvement de sang au pli du coude: dans ce cas la mAECT^3^ après concentration est la plus sensible [14]. Le prélèvement peut aussi être réalisé à partir d'une palpation suivie de ponction ganglionnaire (« état frais »), ou même du liquide céphalo-rachidien (LCR) après ponction lombaire dans le cas du diagnostic de phase, indispensable pour décider du traitement à donner (voir plus bas). Enfin, des outils de diagnostic à partir du derme sont actuellement à l’étude dans le cas de projets de recherche [23].

Comme il n'y a pas de tests sérologiques pour la THA à *T. b. rhodesiense*, la stratégie pour cette forme de maladie est entièrement basée sur le dépistage passif avec une suspicion clinique suivie par la parasitologie.

### Traitement

Pendant très longtemps, le traitement de la THA a reposé sur le triptyque: pentamidine (1^re^ phase THA à *T. b. gambiense)* / suramine (1^re^ phase THA à *T. b. rhodesiense)* / mélarsoprol (2^e^ phase des deux THA). Ce dernier (mélarsoprol ou arsobal) a longtemps été appelé « killer drug » car responsable d'effets secondaires de type encéphalopathies pouvant être fatales, mais il n'y avait pas d'autre choix. Pour la THA à *T. b. gambiense*, un premier progrès significatif a lieu en 2009 lorsque la NECT (nifurtimox eflornithine combination therapy) remplace petit à petit le mélarsoprol pour les malades de seconde phase, mais l'hospitalisation reste obligatoire et les défis logistiques dus à une multitude de perfusions rendent le traitement toujours très contraignant. Depuis 2019 et l'avènement du fexinidazole, on dispose enfin d'un traitement oral traitant les deux phases, ce qui constitue une amélioration significative pour les patients et pour le personnel soignant [8]. Mais il reste quelques limites sérieuses à ce traitement: nécessité d'avoir un repas correct pour assurer l'absorption correcte de la molécule, efficacité limitée pour les patients présentant plus de 100 cellules dans le LCR (d'où l'impossibilité de faire l'impasse sur la ponction lombaire), et impossibilité de traiter des enfants jeunes [15]. La vraie « révolution » dans le traitement de la THA est aujourd'hui la mise au point de l'acoziborole. Ce médicament, d'après les premiers résultats des essais cliniques, permet un traitement oral à prise unique, soigne les deux phases, et ne présente pas d'effets secondaires graves [1]. Il pourrait être envisagé de traiter non seulement les malades, mais d’étendre le traitement à d'autres parts de la population à risque (séropositifs non confirmés, voire plus), si les résultats des essais en cours confirment l'innocuité.

Pour la THA à *T. b. rhodesiense*, les résultats d'un essai clinique en cours avec le fexinidazole sont encourageants, et donnent l'espoir de pouvoir arrêter l'utilisation du mélarsoprol dans cette forme de la maladie également.

### Lutte antivectorielle

En l'absence de vaccin et de chimioprophylaxie, la lutte antivectorielle (LAV) se présente comme le seul moyen de prévention, et de protection des populations contre la piqûre infectante de la tsé-tsé.

Mais rappelons quelques caractéristiques si particulières de cet insecte extraordinaire [24]. Les tsé-tsé sont des insectes diptères d'une famille (les Glossinidae) qui ne comporte qu'un seul genre (*Glossina*) et 31 espèces et sous-espèces. Une douzaine d'entre elles présentent une importance comme vecteur de trypanosomiase humaine ou animale. Actuellement deux d'entre elles, *G.fuscipes* et *G. palpalis*, sont les vecteurs de la THA à *T. b. gambiense.* Les tsé-tsé sont hématophages, et donc vecteurs, dans les deux sexes. Elles pratiquent la « viviparité adénotrophique »: la femelle fécondée nourrit sa larve *in utero* jusqu'au stade L3, qu'elle émet dans le milieu extérieur (larviposition), et qui se transformera dans le sol en pupe (elles sont appelées insectes « pupipares ») dont il émergera un adulte entre 20 et 80 jours après, en fonction des conditions de température et d'humidité. La présence de bactéries symbiontes est indispensable à la nutrition de la larve. Chez les tsé-tsé, seul le premier accouplement est fécondant, et aucune résistance aux insecticides n'a jamais été rapportée jusqu'ici.

Parmi l'arsenal d'outils de LAV, nous nous focalisons sur les systèmes appelés « attractifs toxiques immobiles ». Ces dispositifs basés sur l'attraction visuelle (forme, couleur) et/ou olfactive, ont mené au développement de plusieurs types de pièges et d’écrans basés sur des tissus de couleur bleue qui permettent de piéger ou de tuer ces vecteurs.

**Figure 2:**
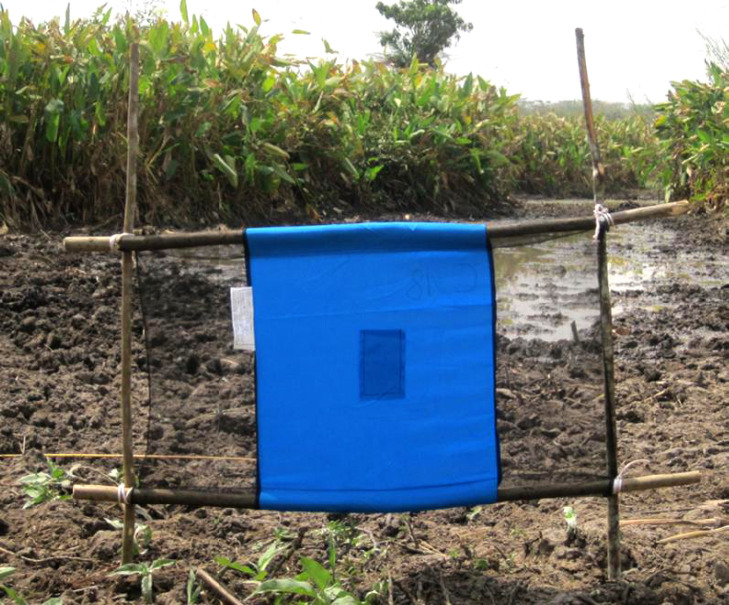
Petit écran ou « tiny target » utilisé pour la lutte anti tsé-tsé dans les foyers actifs de THA en combinaison avec les activités médicales de dépistage et traitement 2016, Bonon, Côte d'Ivoire (crédit photo: Dramane Kaba) Tiny target used for tsetse control in active HAT foci in combination with medical screening and treatment 2016, Bonon, Côte d'Ivoire (photo credit: Dramane Kaba)

En particulier, la mise au point récente des « tiny targets » ou « mini-écrans » [5, 20] (Fig. 2) a permis de disposer d'un outil de lutte très rentable car peu cher, standardisé car fabriqué par un industriel, et disponible et accessible grâce à une donation de son fabricant et des financements dédiés.

Après des années de mise au point, puis d'essais pilotes en Guinée et en Ouganda, ces écrans ont petit à petit été déployés dans les foyers actifs de transmission de THA en Guinée, en Ouganda, mais aussi au Tchad, en Côte d'Ivoire et en République démocratique du Congo (RDC) [7, 13, 16]. À ce jour, on estime que ces petits morceaux de tissu imprégnés de deltaméthrine protègent plus de 4 millions de personnes sur 15 000 km^2^ dans ces 5 pays [18].

C'est cette combinaison d'activités médicales et de lutte antivectorielle, permise par des financements internationaux depuis plusieurs années, coordonnée sur le terrain par les programmes nationaux de lutte et l'ensemble des parties prenantes y inclus les acteurs de la recherche, qui permet l'interruption de la transmission [21].

## Où En Est-On De L’élimination De La Tha, Cible De La Feuille De Route Oms?

**Figure 3:**
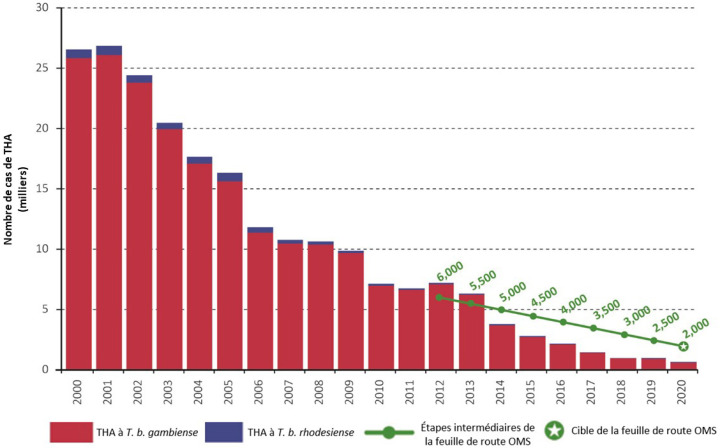
Évolution du nombre de cas rapportés de THA (chiffres OMS) en comparaison avec les cibles de la feuille de route OMS 2021-2030 (source: OMS, 2022 [6]) Evolution of HAT cases compared with WHO 2021-2030 roadmap targets (source: WHO, 2022 [6])

Pour rappel, l'objectif 2020 de la communauté internationale était l’élimination comme problème de santé publique (EPSP), définie par « moins de 1 cas pour 1000 habitants pendant 5 ans consécutifs pour tous les districts sanitaires du pays », et moins de 2000 cas à l’échelle globale. Cet objectif est atteint pour ce qui est de l’échelle globale avec moins de 1000 cas rapportés depuis 2020 (voir Fig. 3), et à l’échelle des pays, plusieurs ont soumis leur dossier d'EPSP qui a été validé par l'OMS [6]. Ainsi, à la date où nous écrivons ces lignes (décembre 2022), le Togo, la Côte d'Ivoire, le Bénin, la Guinée équatoriale, l'Ouganda et le Rwanda sont dans ce cas. Plusieurs autres préparent actuellement leur dossier de validation. Enfin certains pays présentent toujours trop de cas et de transmission, ou pas assez d'information pour rédiger leur dossier. Nous nous attarderons sur deux cas emblématiques et différents, la Côte d'Ivoire et la Guinée.

La Côte d'Ivoire a été validée par l'OMS comme EPSP, et a présenté un plan avec des activités visant l’élimination avec interruption de la transmission d'ici 2025.

En Guinée la situation est différente, car beaucoup plus de cas étaient rapportés ces dernières années, avec en sus les crises sanitaires récentes comme Ébola puis le SARS-CoV-2 qui ont eu des conséquences dramatiques sur les activités médicales et l'accès des équipes médicales aux populations, y compris pour la THA [2, 3]. Mais il faut souligner les résultats absolument remarquables de cette combinaison d'activités médicales et de LAV qui permettent une baisse drastique de la transmission et autorisent la Guinée à rédiger son dossier EPSP pour 2023.

L'objectif de la feuille de route de l'OMS, concernant la THA à *T. b. gambiense*, est l’élimination avec interruption de la transmission d'ici 2030 et bon nombre de pays s'inscrivent dans cette dynamique et cet objectif, qu'il conviendra d'accompagner au mieux.

## Défis Pour Une Élimination Durable De La Tha

Il n'est pas question ici de donner une liste exhaustive de tous les défis existants pour atteindre une élimination avec interruption de la transmission qui se voudrait durable et constituerait *in fine*, une, si ce n'est LA meilleure prévention à long terme contre la ré-émergence. Aussi, nous prenons quelques exemples de défis qui se posent à la recherche, tout en reconnaissant que beaucoup d'autres relèvent de domaines sur lesquels la recherche n'a pas de prise. Cependant nombre de ces défis tournent autour, encore et toujours de la notion d'accès, si intimement liée aux MTN.

Parmi les défis « recherche », la question du portage chronique de parasites, donc des réservoirs (qu'ils soient humains ou animaux), est probablement l'une des plus prégnantes, pour autant que tout passage de parasite du compartiment animal vers l'homme *via* le vecteur, même statistiquement « rare », serait susceptible d'annihiler l'interruption de la transmission [17, 25]. C'est aussi l'une des raisons qui ne nous fait pas employer la notion d’éradication de la THA. Liées à cette question, les notions de démonstration de l'absence (par opposition à la présence), de « glocal » (mélanges d’échelles globales et locales d'intervention), de nouveaux diagnostics adaptés à cette question, de développement d'outils de LAV non polluants, sont des exemples de défis auxquels la recherche doit encore apporter des réponses, dans un contexte où l'acoziborole peut s'avérer un « game changer » décisif.

Mais reconnaissons que pour parvenir à l’élimination, et la rendre durable, d'autres défis existent sur lesquels les chercheurs n'ont peu ou pas de prise directe, même si l'on peut considérer qu'il est de leur devoir de s'y intéresser, voire de s'y impliquer. Ainsi, la notion d'accès est centrale: accès aux diagnostics, accès aux traitements, accès à la lutte antivectorielle. Rappelons que pour la THA, tous ces outils font l'objet de donations ou de subventions, mais jusqu’à quand? Quelle certitude pour les années à venir?

Autre défi, et non des moindres: comment faire en sorte que les programmes nationaux des ministères de la santé continuent à mobiliser des ressources humaines sur une maladie qui, non contente de répondre à la définition d'une maladie déjà qualifiée de « négligée », est de plus en voie d’élimination et ne constitue plus un problème de santé publique? Comment garder les bailleurs de fonds, les chercheurs, les partenariats public/privé, et point le plus important, les agents de santé spécialisés impliqués dans ce contexte?

Et pourtant, aller « au bout » de cette élimination ne constitue-t-il pas l'exemple à suivre, au fond la meilleure garantie sur le long terme, d'une prévention de toute ré-émergence pour la THA comme pour la plupart des maladies qualifiées d’émergentes ou ré-émergentes [22]?

## Contribution Des Auteurs

Tous les auteurs ont contribué à la production et à l'analyse des données de cette étude. La première version du manuscrit a été rédigée par Philippe Solano et tous les auteurs ont commenté les versions suivantes du manuscrit. Tous les auteurs ont lu et approuvé le manuscrit final.

## Remerciements

Les auteurs tiennent à remercier leurs institutions, ainsi que tous les contributeurs à la lutte contre la THA, quel que soit leur statut. Nous remercions en particulier pour leur contribution financière actuelle l'Union européenne (programme EDCTP2) et la Bill and Melinda Gates Foundation.

## Liens Intérêts

Les auteurs ne déclarent aucun lien d'intérêt.
